# Processing of Emotions in Functional Movement Disorder: An Exploratory fMRI Study

**DOI:** 10.3389/fneur.2019.00861

**Published:** 2019-08-14

**Authors:** Petr Sojka, Jan Lošák, Martin Lamoš, Martin Bareš, Tomáš Kašpárek, M. Brázdil, M. Baláž, Miroslav Světlák, J. Kočvarová, J. Fialová

**Affiliations:** ^1^Department of Neurology, Faculty of Medicine, Masaryk University and St. Anne's University Hospital Brno, Brno, Czechia; ^2^Department of Psychology and Psychosomatics, Faculty of Medicine, Masaryk University and University Hospital Brno, Brno, Czechia; ^3^Department of Psychiatry, Faculty of Medicine, Masaryk University and University Hospital Brno, Brno, Czechia; ^4^Multimodal and Functional Neuroimaging, CEITEC, Masaryk University, Brno, Czechia; ^5^Department of Public Health, Faculty of Medicine, Masaryk University, Brno, Czechia

**Keywords:** emotion regulation, functional neurological disorder, neuroimaging, alexithymia, precuneus

## Abstract

**Background:** Affective dysregulation and impaired cognitive control are implicated in the pathology of functional neurological disorders (FNDs). However, voluntary regulation of emotions has seldom been researched in this group of patients. We hypothesized that patients with FNDs use inefficient voluntary emotion regulation strategies and regulate emotional reactions via increased motor activation.

**Methods:** Fifteen patients with functional movement disorder (FMD) and fifteen healthy subjects matched by age, sex, and education underwent an emotion regulation task in fMRI. For stimuli, we used neutral and negative pictures from the International Affective Picture System. There was no restriction on their emotion regulation strategy. Both patients and healthy subjects were asked about the strategies they had used in a post-scanning interview. Participant levels of depression, trait anxiety, and alexithymia were assessed.

**Results:** There were no significant differences in the emotion regulation strategies used by patients and healthy subjects, nor in levels of reported alexithymia and depression. However, patients showed increased activation in several brain areas when observing negative pictures, notably in the post-central gyrus, precuneus, posterior cingulate cortex (PCC) and cerebellar vermis, and also in their emotion regulation condition, particularly in the precuneus and post-central gyrus. Alexithymia was negatively associated with left insular activation during the observation of unpleasant stimuli only in the patient group.

**Conclusions:** Our findings may implicate areas associated with self-referential processing in voluntary emotional regulation and lower emotional awareness as having a role in patients with functional movement disorders. However, our findings must be replicated with larger sample.

## Introduction

A functional neurological disorder (FND) is a condition in which a patient has neurological symptoms in the absence of neurological disease. FND spans a variety of symptoms such as non-epileptic seizures, abnormal movements (gait disorders, tremor, dystonia, etc.), weakness, and sensory symptoms. Even after more than 100 years of research interest, a pathological mechanism underlying FND is still a subject of debate.

A growing body of neuroimaging evidence supports the notion that abnormal emotional processing is a key factor in the etiology of functional neurological symptoms (1, 2). Task-based neuroimaging studies show limbic and paralimbic hyperactivation (3–5), abnormal limbic-motor circuit connectivity (4–6), and altered activation of several prefrontal regions in emotion processing tasks in various groups of patients with FND (3, 6–9). These findings suggest that unregulated emotional reactions may exert an abnormal influence on the motor system.

Following Janet (10) and Ludwig (11), contemporary cognitive models afford a central role in attentional processes in the etiology of FND (12, 13). There is evidence that both higher-level endogenous attention control and lower-level automatic attentional orienting are impaired in FND. Deficits in voluntary attentional disengagement from emotionally neutral stimuli (14) and the abnormal automatic (pre-conscious) allocation of attention to facial affect (15), specifically to threat-related facial affect (16), were found in FND patients. Moreover, avoidance learning of negative stimulus was shown to be impaired in FND (17). These findings demonstrate that FND patients show diminished cognitive processing in emotional contexts.

Taken together, the emerging model that could help in understanding FND combines higher-order (attention, cognitive control) and bottom-up limbic processes (limbic hyperactivity, limbic-motor connectivity) interacting to influence motor control (18). Despite research evidence of impaired cognitive processing in emotional contexts, only minor research focus has been given to voluntary emotional regulation in FND. Voluntary emotional regulation refers to intentional up or down-regulation of an emotional reaction and it is contrasted to automatic (non-conscious) voluntary regulation such as an avoidance of stimulus (19). To our knowledge, only one neuroimaging study utilizing magnetoencephalography examined voluntary emotional regulation in FND, with a finding of reduced fronto-cortical, but enhanced sensorimotor involvement in emotion regulation efforts (20). This finding suggests that the patients had lower cognitive control over emotional stimuli and may activate different (“less cognitive”) emotion regulation strategies, reflected in sensorimotor network activation even though the authors of the study restricted the task to cognitive reappraisal of emotional stimuli. Opitz et al. (21) point to the fact that people in laboratory settings are likely to use whichever emotion-regulation strategies work best for them even when they have been trained and instructed to use one specific strategy.

Given the abnormal attention to emotional stimuli reported in FND, patients may use attentional deployment (e.g., avoidance) or emotional reaction suppression as ways to regulate emotional reactions. For example, Ferri et al. (22) found that diverting attention from unpleasant emotional stimuli also predominantly activates the parietal regions in healthy subjects, similarly to findings in FND reported by Fiess et al. (20). Suppression of emotion-expressive behaviors was shown to reduce negative emotional experiences but sustain elevated responses in the amygdala (23); this finding is also relevant to FND, as failure to habituate the amygdala in response to emotional stimuli was observed in this group of patients (5). We hypothesize that the hyperarousal and diminished habituation of the amygdala documented in FND may be associated with inefficient voluntary emotional regulation. We therefore conducted an exploratory study aimed at identifying the natural emotion regulation strategies [“spontaneous regulation”; (24)] employed by FND patients and to explore brain activation related to the voluntary emotion regulation in this group of patients.

## Materials and Methods

### Participants

For the purpose of the study, we selected a sub-population of FND with predominant motor signs to ensure relative sample homogeneity. Fifteen adult patients with clinically definite functional movement disorder (FMD), diagnosed based on established clinical criteria (25), were recruited from the neurology clinic at Masaryk University together with fifteen healthy controls recruited from the general population and matched with the patients by sex, age, and education. The sample size was determined on the basis of previous neuroimaging research utilizing emotional stimulation in FMD patients (e.g., (5), *N* = 16; (6), *N* = 12; (7), *N* = 10; (8), *N* = 12) and also on the basis of the emotion-regulation study that reported sample sizes of 18 per group as sufficient to gain statistical power of 80% (26). Only patients with symptoms persisting for more than 2 years and healthy volunteers with no previous neurological or psychiatric symptoms were included in the present study. Demographic and neurological data were recorded, and depression, trait anxiety, and alexithymia, defined as restricted access to emotional information (27), were evaluated. Patient clinical characteristics are summarized in Table 1. All participants gave their written informed consent and received monetary compensation. The study was approved by the ethics committee of Masaryk University and St. Anne's Hospital.

**Table 1:** Clinical and demographic characteristics of functional movement disorder patients.

**Patient**	**Clinical signs**	**Gender**	**Age**	**Illness duration**	**Comorbid affective disorder**	**Medication**
1	Dystonia	F	52	4	Depressive syndrome, panic attacks	Venlafaxine
2	Body stiffness and spasms	F	46	4	Depressive syndrome	Oxazepam and SSRI
3	Tremor of right hand	F	56	11	None	None
4	Right leg weakness	M	20	3	None	None
5	Quadriparesis	F	59	2	None	None
6	Gait disorder	F	63	5	None	None
7	Myoclonus	F	61	15	None	None
8	Tremor of right hand	F	51	2	None	None
9	Tremor of both hands, gait disorder	F	31	2	Depressive syndrome	None
10	Tremor, abnormal movements of chin	F	22	3	None	None
11	Myoclonus	M	20	2	None	None
12	Gait disorder	F	29	6	None	Agomelatine
13	Weakness of both legs, non-epileptic seizures	M	41	8	Depressive syndrome, anxiety	Mirtazapine
14	Tremor and dystonia	M	24	2	None	None
15	Weakness of both arms, non-epileptic seizures	F	21	4	None	None

*Comorbid affective disorder refers to presence of clinically significant symptoms of anxiety or depression assessed by a clinical psychologist. F, female; M, male. Age and illness duration is displayed in years*.

### Self-Report Measures

#### Beck Depression Inventory

Since depression is a common comorbidity in FND (28), we included the Beck Depression Inventory, second version [BDI-2; (29)] to control for the influence of reported depression on emotional regulation. BDI-2 is a widely used 21-question multiple-choice self-report inventory with high internal consistency (30) that measures characteristic attitudes and symptoms of depression.

#### State-Trait Anxiety Inventory

The trait scale from the State-Trait Anxiety Inventory [STAI-T; (31)] was used to measure the trait of anxiety. The scale consists of 20 statements that are rated on a four-point Likert scale. STAI-T scores were used as a covariate to control for the effects of anxiety, which is commonly associated with the tendency to experience somatic symptoms (32).

#### Toronto Alexithymia Scale

Alexithymia has been shown to limit emotional regulation in healthy subjects as well as in patient samples (33, 34). FMD patients have been found to be significantly more alexithymic than patients with organic motor disorders and healthy controls (35), so we included a measure of alexithymia in the study as a factor potentially contributing to emotional dysregulation in FMD. The Toronto Alexithymia Scale [TAS-20; (36)] is a well-validated and commonly used measure of alexithymia. TAS-20 is a multidimensional self-report instrument with a three-factor structure: difficulty identifying feelings, difficulty describing feelings, and externally oriented thinking. Items are rated using a 5-point Likert scale and the total score of alexithymia is calculated as a sum of the three subscales.

### Emotion-Regulation Task

#### Stimuli

Emotional and neutral pictures from the International Affective Picture System [IAPS; (37)] and emotionally negative pictures were selected with an emphasis on scenes with threat-related content (e.g., frightened people, weapons, attacks, surgical procedures), as threat sensitivity was repeatedly observed in patients with functional neurological symptoms (7, 16, 38). The selected negative pictures had mean normative valence ratings of 2.54, and mean arousal ratings of 5.66. Selected neutral images had mean valence ratings of 5.10 and mean arousal ratings of 3.26 (see Supplementary Material).

#### Task Design

**Figure 1:**
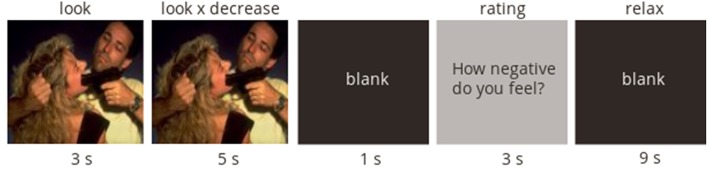
Scheme of the task design. Neutral and negative pictures were first presented with a “look” instruction for 3 s. In neutral pictures and half of the negative pictures the “look” instruction remained on the screen for another 5 s. In the other half of negative pictures, the instruction “look” changed into “regulate” instruction after initial 3 s of picture viewing.

At the start of each trial, a picture was presented with an instruction word displayed below the picture (“look”; 3 s); the instruction remained for the next 5 s for neutral pictures and negative pictures without regulation, or the instruction changed to the regulation instruction (“decrease,” 5 s) for negative pictures with regulation. The presentation of each picture was followed by 1 s of blank screen, followed by a self-reported rating of the strength of the negative effect (on a scale from 1 to 4, where 1 was labeled “not at all” and 4 was labeled “very much”; 3 s), finally the word “relax” appeared on a blank screen for the rest of the trial (9 s). The task design follows a methodology used by Jackson et al. (39) and is depicted in Figure 1. Responses were made on a 4-button box using the participant's dominant (right) hand. The combinations of instructions and pictures produced three trial types: decrease negative (regulation), look negative (non-regulation), and look neutral (non-emotional). A total of 66 trials (22 of each trial type) were administered in pseudo-randomized order with the constraint that no more than two of any trial type or picture type followed each other sequentially. The task was presented with E-Prime (Psychology Software Tools Inc, Pittsburgh).

### Procedure

Pre-MRI, participants completed questionnaires and were familiarized with the task by practicing several rounds of the task with a different set of IAPS pictures than those used with the MRI. The participants were not given any specific instructions for emotion regulation strategies to use but were only asked to try to down-regulate an emotion that might occur in the reaction to the presented pictures. The participants were also informed about a post-scanning interview about the emotion regulation strategies they applied during the task. In the post-scanning interview, the responses of participants were coded into three categories based on a process model of emotional regulation (40). The categories were: attentional deployment, cognitive reappraisal, and emotional response modulation. Attentional deployment refers to focusing on non-emotional details of a picture or focusing on one's own thoughts unrelated to a picture. Cognitive reappraisal involves reinterpreting the meaning of the emotional stimulus (e.g., a gun on a picture is reinterpreted as a mock-up weapon). Emotional response modulation refers to efforts to modify an emotion after it has been fully generated; volitional inhibition of verbal and behavioral expressions of emotions is the most frequent form of this strategy.

### MRI Data Acquisition

MRI scanning was performed using a 3-Tesla whole-body MRI scanner SIEMENS MAGNETOM Prisma (Siemens Medical Systems, Erlangen, Germany) at the Central European Institute of Technology, Brno, Czech Republic. At the beginning, a high-resolution anatomical T1-weighted scan was acquired with the following parameters: magnetization-prepared rapid gradient-echo (MPRAGE) sequence [repetition time (TR) = 2,300 ms, echo time (TE) = 2.33 ms, flip angle (FA) = 8°, voxel size 1.00 × 1.00 × 1.00 mm, slice thickness 1.00 mm, matrix 240 × 224 × 224]. Subsequently, whole brain functional measurement was performed by multiband acquisition with the parameters: TR = 642 ms, TE = 35.0 ms, FA = 47°, voxel size 3.3 × 3.3 × 3.5 mm, 40 sagittal slices, field of view 210 × 210 mm. The total number of volumes was 2,175.

### Analysis of Self-Report and Behavioral Data

A statistical analysis was performed using Python numerical and statistical libraries. The variables were first tested for normality using the Shapiro-Wilk test. The variables that were not normally distributed were log-transformed. For continuous data, a two-way analysis of variance (ANOVA) was used to test for differences across the two groups and three task conditions with *post-hoc* Bonferroni pairwise comparisons when significant. The χ^2^-test was used for categorical data and the Pearson correlation coefficient (r) was used to examine potential associations between behavioral and neuroimaging findings. Bonferroni correction was applied to correct for multiple comparisons.

### Analysis of fMRI Data

MRI data were processed and analyzed using SPM12 (Welcome Department of Cognitive Neurology, London, UK). The preprocessing of fMRI images included realignment to correct for head movements. Subsequently, co-registration of functional and anatomical images and interpolation in time were performed, followed by the spatial normalization into the stereotactic Montreal Neurological Institute (MNI) space and spatial smoothing (isotropic Gaussian kernel of 8 mm full-width at half-maximum). The motion related artifacts were regressed from the data by setting up a general linear model design using 24 motion parameters (41).

In the first level of analysis, six separate regressors in the generalized linear model were specified for fMRI responses to the initial negative or neutral stimulus viewing; further attending to neutral or negative stimulus; regulation of negative stimulus; and blank screen. Individual statistical parametric maps were calculated for the following contrasts of interest in order to investigate BOLD signal changes: negative-look vs. neutral-look contrast for the effect of emotional stimuli (initial negative or neutral stimulus) and negative-regulate vs. negative-look contrast for the effect of emotion regulation (regulation of negative stimulus or further attending to negative stimulus). Values for both contrasts were subjected to second-level analysis.

To obtain the second level between-group z-statistics, statistical maps were thresholded at a z value > 3.2 (cluster forming threshold, *p* < 0.001) and a cluster-corrected FWE correction threshold (*p* < 0.05) was calculated using Gaussian random field theory. We performed an ANCOVA to test for differences in the two contrasts. Age, sex, BDI, and STAI were used as nuisance variables. Due to the exploratory nature of the study, we report both significant clusters (*p* < 0.05) after FWE correction and uncorrected results with *p* < 0.001 threshold the cluster level.

## Results

### Participant Characteristics and Behavioral Findings

In total, the study had fifteen patients (eleven females) with a mean age of 39.7 (SD = 16.5) years. Demographic and clinical characteristics are provided in Table 1. Fifteen HCs (eleven females) had a mean age of 40.3 (SD = 15.9) years. Patients and HCs did not differ significantly with respect to age, gender, or education. As expected, more patients used psychotropic medication. Patients had higher scores than HCs on both the STAI-T and BDI-2 scales. There was no significant difference in TAS-20 between patients and HCs, suggesting low alexithymia in our sample (see Table 2 for further details).

**Table 2:** Descriptive statistics for the self-report and behavioral variables.

**Variable**	**FMD**	**HC**	**Statistics**
Mean age (SD)	39.73 y (16.54)	40.27 y (15.88)	*t*_(28)_ = 0.09, *p* = 0.93
Mean symptom duration	4.87 y (3.80)		
BDI-2	22.93 (12.92)	16.29 (8.40)	*t*_(28)_ = 1.61, *p* = 0.12
STAI-T	40.80 (12.24)	32.86 (7.48)	*t*_(28)_ = 2.12, *p* = 0.04
TAS-20	46.33 (12.55)	41.71 (12.42)	*t*_(28)_ = 0.10, *p* = 0.33
Neutral-look	36.40 (13.10)	33.13 (11.63)	
Negative-look	38.67 (13.60)	43.60 (11.61)	
Negative-regulation	39.13 (13.94)	43.47 (13.72)	
Attentional deployment	11 (36.7%)	9 (30%)	
Cognitive reappraisal	4 (13.3%)	5 (16.7%)	
Reaction modulation	0 (0%)	1 (3.3%)	

*BDI-2, Beck depression inventory; STAI-T, trait scale from the State-Trait Anxiety Inventory; TAS-20, Toronto alexithymia scale*.

To test possible group and task condition differences in negative emotion rating induced by stimuli (IAPS pictures), we conducted a two-way ANOVA with group (patients, HCs) and task condition (neutral-look, negative-look, negative-regulate) as between-subject factors. There was no interaction effect [*F*_(2, 84)_ = 0.93, *p* = 0.398], nor mean effect of group [*F*_(1, 84)_ = 0.670, *p* = 0.414], but ANOVA revealed a significant main effect of the task condition on negative emotion rating [*F*_(2, 84)_ = 3.62, *p* = 0.031]. *Post-hoc* comparisons using Tukey's HSD test indicated that the mean score for the rating in the negative-look condition (M = 41.13, SD = 12.68) was significantly different than the rating in the neutral-look condition (M = 34.8, SD = 12.29). However, there was no significant difference between neutral-look and negative-regulate, nor between negative-look and negative-regulate conditions. These results indicate that the induction of negative emotional experience was successful in both groups, but the down-regulation of emotion was unsuccessful. Moreover, the compared groups (FMD vs. HC) do not differ in the task conditions.

We also compared the frequency of emotion regulation strategies reported by patients and HCs in the post-scanning interview. The strategies were clustered into three categories: attentional deployment, cognitive reappraisal, and emotion-response modulation. The strategies used were not significantly different between HCs and patients [$\chi_{\left(2\right)}^{2}$ = 1.311, *p* = 0.519]; both groups used attentional deployment as most preferred emotional regulation strategy (see Table 2).

### Imaging Findings

No data were discarded due to motion-related or other artifacts. With the negative-look vs. neutral-look contrast (effect of emotion induction), regional differences were found between the FMD vs. HC group when controlling for depression and anxiety but only at a lower statistical threshold (uncorrected *p* < 0.001). Notably, the FMDs showed increased activation in left postcentral gyrus, right superior parietal lobe/precuneus, left PCC, and right cerebellar cortex. We also observed decreased activation in the bilateral insula in FMD patients as compared to HCs. There were no significant activations in the reversed comparison.

In the negative-regulate vs. negative-look contrast, no differences between the FMD and HC group survived FWE correction; however, FMD patients showed increased activation in two regions at a lower statistical threshold (*p* < 0.001 uncorrected). The effect of emotional regulation specific to FMD patients was underpinned by increased activity in the right superior parietal lobe/precuneus and left postcentral gyrus (see Table 3).

**Table 3:** fMRI results for the negative-look (NegL) > neutral-look (NeuL) and negative-regulate (NegR) > negative-look (NegL) contrasts for the functional movement disorder (FMD) > healthy control (HC) comparison.

**Comparison**	**Contrast**	**Cluster mm^**3**^**	**t^**+**^**	**MNI**			**Side**	**Region**	**Talairach**
				**x**	**y**	**z**			
FMD>HC^*^	NegL>NeuL	72	4.72	−22	−32	76	L	Post-central gyrus	
		192	4.15	28	−42	64	R	Precuneus	BA 7
		152	4.12	−12	−34	44	L	Post-cingulate gyrus	BA 31
		176	4.06	−40	−8	20	L	Insula	
		144	4.04	42	−14	−8	R	Insula	
		48	3.76	20	−58	−50	R	Cerebellar lobule VI	
FMD>HC^*^	NegR>NegL	304	4.35	16	−70	64	R	Precuneus	BA 7
		16	3.85	−22	−32	76	L	Post-central gyrus	

*There was no significant activation in the opposite direction*.

* *p <0.001 uncorrected*.

+ *The t value indicates the peak statistical value for the cluster*.

*MNI, Montreal Neurological Institute*.

**Figure 2:**
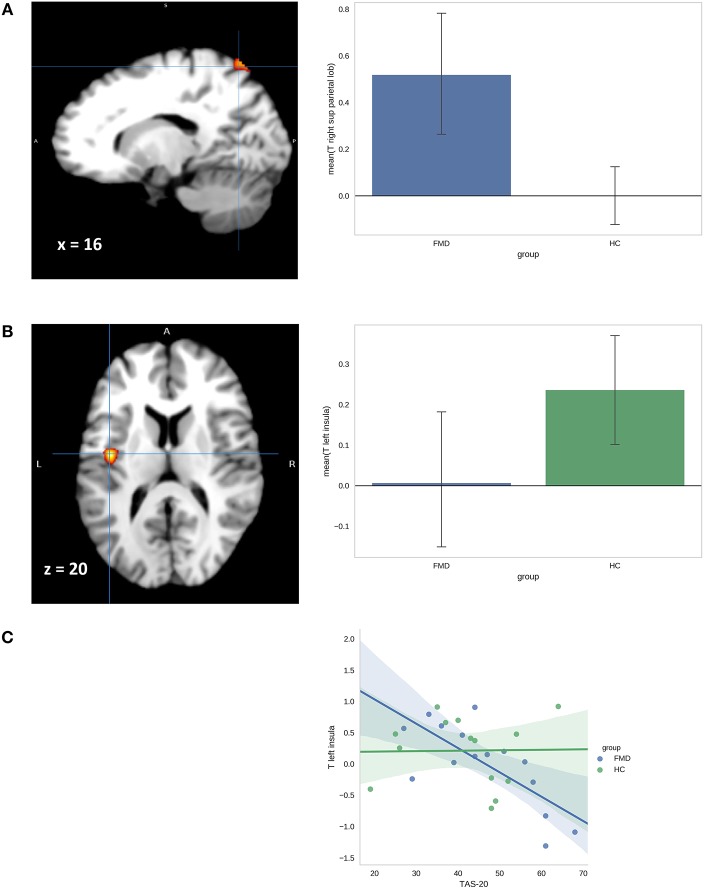
**(A)** Activation map and adjacent barplot demonstrate group differences in activation of the left superior parietal lobus in the negative-regulate>negative look contrast. **(B)** Activation map and adjacent bar-plot shows group differences in the left insula activation in the contrast negative-look>neutral-look. **(C)** Scatterplot shows the relationship between *t*-values for the negative-look>neutral-look contrast in left insula and TAS-20 scores for FMD patients and HCs.

Despite the moderate levels of alexithymia in both FMD and HC groups, we tested for potential differences in association between scores in TAS-20 and activations in FMD and HCs in both reported contrasts, as alexithymia has been shown to influence emotional regulation. Although the TAS-20 scores did not vary between groups (Table 2), alexithymia was found to differentially influence activation in the left insula in the FMD and HC groups. FMD patients exhibited decreased activation in the left insula in negative-look vs. neutral-look contrast with increasing levels of alexithymia (Figure 2); *r* = −0.74, *p* = 0.0018 (with *p* < 0.003 threshold after Bonferroni correction for multiple tests). The correlation between alexithymia and left insula activation was not significant in HCs; *r* = 0.016, *p* = 0.96. No other significant associations were found between TAS-20 scores and task-related brain activations.

## Discussion

The current study examined neural activation associated with uninstructed voluntary emotional regulation in FMD patients. We also examined the association between alexithymia and the ability to regulate emotions in FMDs. We successfully induced emotional response in the participants, but emotion regulation did not decrease negative feelings across the groups. There were no differences in negative emotional experience induced by stimuli between FMDs and HCs. We observed several differences in brain activations between FMDs and HCs but only on a more liberal statistical threshold. In comparison to HCs, FMD patients showed increased activation in the right superior parietal lobe/precuneus and in the left post-central gyrus during emotion regulation attempts (relative to observing negative stimuli). Increased activation in the right superior parietal lobe/precuneus and in the left post-central gyrus was observed in FMD also during observation of negative stimuli compared to neutral pictures.

Bilateral superior parietal lobe/precuneus activation has been documented during focusing on both arousing and non-arousing regions of unpleasant images in healthy subjects and is therefore implicated in the emotion regulation strategy of attentional deployment (22). Both FMD patients and HCs in our study used attentional deployment as the most preferred emotion regulation strategy with no significant differences between groups. However, only the FMD group showed increases in precuneus activation that was also present during exposure to negative stimuli without regulation instruction. The precuneus is considered to be a part of the default mode network and has been associated with self-monitoring and consciousness (42). Activity in medial parts of the precuneus has been elicited in tasks involving motor imagery (43, 44) and episodic memory retrieval (45, 46). In FND, activation in the precuneus was reported during attempts to move in functional paralysis with selective changes in functional connectivity of the motor cortex with the precuneus during functional paralysis (47). We therefore suggest that the observed activations in the right precuneus together with the left postcentral gyrus may reflect implicit emotional processing rather than voluntary attention control (e.g., focusing on non-arousing aspects of pictures). Neither option can be ruled out; this should be a subject of future research. Moreover, the precuneus has been implicated in dissociative phenomena associated with abnormal self-awareness. Nicholson et al. (48) found increased resting-state precuneus-amygdala activation in the dissociative subtype of posttraumatic stress disorder and increased precuneus activation was also reported in hypnotically induced limb paralysis (47). Our findings may further corroborate dissociation theories highlighting a role of self-monitoring and self-related mental representations during voluntary efforts in FND.

Several brain areas were differentially activated in FMDs and HCs while observing unpleasant pictures as compared to neutral pictures. In addition to increased activation in the right precuneus and left post-central gyrus, we also observed increased activation in the left PCC and right cerebellar lobule VI in FMD patients as compared to HCs. Increased PCC activation was observed in FND in the emotional induction paradigm (7), during motor preparation in functional paralysis (47) and also in functional tremor (49). Moreover, the PCC was implicated in self-reflection (50) and in the integration of emotion and memory (51). Specifically in FND, Blakemore et al. (7) interpreted increased PCC activation as an abnormal access to self-relevant information in memory which can further modulate action readiness. Furthermore, we observed increased cerebellar activation in right lobule VI in the FMD patients. Lobule VI has been associated with processing aversive stimuli in the form of activating motor plans associated with action preparedness (52). Taken together, observed increased PCC and cerebellar activation may thus be indicative of instinctive behavioral responses to threat-related information in FND, as was formulated by Kretschmer (53).

Interestingly, we found decreased insular activity during exposure to negative stimuli in FMD patients as compared to HCs. Although there were no differences in levels of alexithymia between FMDs and HCs, we found that the activation in the left insula was negatively correlated with levels of alexithymia only in the FMD group. Functional neuroimaging studies employing emotionally arousing stimuli such as disgusting, frightening, or sexual pictures have consistently reported activation in the insula in healthy subjects (54). Furthermore, alexithymia is commonly seen in patients with functional deficits of the insular cortex such as frontotemporal dementia (55) and autism (56), and under activation of the insula has been associated with deficits in emotional awareness (57). Taken together, our findings may point to low emotional awareness in FMD patients and their tendency to react to unpleasant stimuli more physically, as reflected in the increased cerebellar and PCC activation in FMD patients during exposure to negative stimuli.

Contrary to previous research (3–5), we did not observe amygdala hyperactivation in FMDs in emotion induction contrast. However, recent research studies utilizing IAPS stimuli also failed to find differential response in amygdala between healthy subjects and FMD (7), functional dystonia (8) and functional tremor patients (9). Similar to our findings, Espay et al. (8) reported decreased right insular activation in response to emotional stimuli together with decrease in activation in bilateral precuneus in functional dystonia compared to healthy subjects. Research studies reporting amygdala hyperactivation in FND used facial expressions as stimuli to induce the activation (3–5). The amygdala routinely responds to novel stimuli (58, 59) and Somerville and Whalen (60) noted that amygdalar response to facial stimuli such as Ekman faces (61) may represent reaction to novelty as these facial expressions are not genuine and therefore not typically seen in daily life. Hyperactivity of amygdala observed in FND patients may thus represent abnormal reaction to novel stimuli but may not be related to environmentally meaningful emotional responses such as defense reactions.

Several limitations of the present study have to be addressed. First, the presented results need to be interpreted cautiously due to relatively small sample size. However, small sample sizes are common in neuroimaging research of FND due to difficulties in patient recruitment and aberrant movements often precluding MRI measurement. Due to the limited sample size we could not cluster patients and healthy controls according to the emotion regulation strategy they used, and we were unable to determine the effects of emotion regulation strategy on the patterns of brain activation. Future studies could focus on comparing different emotion regulation strategies within a group of FND patients (such as emotional suppression or avoidance of emotional stimuli). The depression scores in our control group were relatively high, with average scores in the range of mild depression. This result might indicate a presence of emotional dysregulation in HCs; however, over-reporting of depression in BDI-2 is also possible. The influence of depression on the results is not probable as there is a non-significant difference in BDI-2 between HCs and FMDs, and we also controlled for the effect of depression in the neuroimaging analysis. We did not assess a level of trauma in the participants of our study. The nature of chosen emotion stimuli (e.g., violent scenes) may evoked past traumatic experiences especially in the FMD group, where trauma rates are found to be higher than in the general population (62). Evoked traumatic experiences may consequently influence emotion-regulation (63) and reporting in the post-scanning interview. Even though we were careful to make sure the participants understood the task, a realization of how one regulates emotion is inherently a difficult task without a previous intensive training. Moreover, given a low emotional awareness observed in FND (35, 64), a reliability of self-reported emotional experience (depression, alexithymia, negative feeling reports) is problematic in this group of patients (24). Taken together, failure to detect differences in self-reports between FMDs and HCs may be caused by small sample size but also by patients' difficulties providing reliable reports of their emotional experience and regulation process. Finally, we included patients with a broad range of functional movement symptoms which may preclude isolating a pathophysiological mechanism underlying specific symptom presentation (8, 9). However, Edwards (65) pointed out that functional neurological symptoms commonly co-occur and a unifying pathophysiology is therefore likely across different functional symptom phenotypes. For this reason, the division of FND based on prevalent motor symptoms may be seen as rather artificial. Our results, if replicated, point to a more general mechanism underlying abnormal emotional processing in FND patients.

## Conclusion

Our study suggests an abnormal involvement of areas implicated in self-referential processing during voluntary emotional regulation efforts and limited access to emotional experience in FMD patients. Our results may indicate that emotional reactions to negative stimuli are inaccessible for conscious processing due to low emotional awareness and the implicit emotional reactions may therefore pose a difficulty for voluntary emotional regulation efforts. As a result, more bodily emotional regulation processes such as aberrant movements ma develop instead in FMD patients in order to decrease accumulated arousal. A similar view was postulated by Janet (10): “Action, by becoming unconscious in hysterics, by separating from consciousness…assumes an appearance that recalls the action of visceral muscles…” (s. 137). However, the findings presented in this study have to be considered preliminary due to the small sample size and the liberal statistical threshold used in neuroimaging analyses. Future studies should employ experimental tasks probing emotional awareness to further elucidate the role of (un)conscious emotional processing in affective dysregulation in FNDs.

## Data Availability

The datasets generated for this study are available on request to the corresponding author.

## Ethics Statement

This study was carried out in accordance with the recommendations of with written informed consent from all subjects. All subjects gave written informed consent in accordance with the Declaration of Helsinki. The protocol was approved by the Institutional Review Board of the University Hospital of St. Anne and Institutional Review Board of the Masaryk University, Brno, Czech Republic.

## Author Contributions

The first draft of the paper was written by PS. ML designed the emotion regulation task and JL performed MRI analyses. MBar, TK, MBrá, MBal, and MS critically revised and commented on the manuscript and figures. JK and JF recruited patients and healthy controls. All the authors substantially contributed to and have approved the final manuscript.

### Conflict of Interest Statement

The authors declare that the research was conducted in the absence of any commercial or financial relationships that could be construed as a potential conflict of interest.

## Supplementary Material

Table S1 Pictures from IAPS selected for the task.
