# Supplementary material for: Processing of Emotions in Functional Movement Disorder: An Exploratory fMRI Study
Source: Front Neurol. 2019 Aug 14;10:861. doi: 10.3389/fneur.2019.00861 (PMC6703143; doi:10.3389/fneur.2019.00861)
Supplement: Table S1 — Pictures from IAPS selected for the task. [file Data_Sheet_1.DOCX]

| **neutral** | **negative** | **negative - regulation** |
| --- | --- | --- |
| 2038.jpg | 2205.jpg | 2053.jpg |
| 2102.jpg | 2661.jpg | 2141.jpg |
| 2191.jpg | 2691.jpg | 2683.jpg |
| 2214.jpg | 2703.jpg | 2700.jpg |
| 2381.jpg | 2717.jpg | 2710.jpg |
| 2383.jpg | 2718.jpg | 2750.jpg |
| 2393.jpg | 2900.jpg | 2799.jpg |
| 2396.jpg | 3181.jpg | 3120.jpg |
| 2397.jpg | 3215.jpg | 3180.jpg |
| 2446.jpg | 3220.jpg | 3191.jpg |
| 2480.jpg | 3225.jpg | 3216.jpg |
| 2495.jpg | 3301.jpg | 3300.jpg |
| 2499.jpg | 3350.jpg | 3302.jpg |
| 2514.jpg | 6350.jpg | 6313.jpg |
| 2518.jpg | 6550.jpg | 6540.jpg |
| 2570.jpg | 6570.jpg | 6560.jpg |
| 2595.jpg | 8480.jpg | 8485.jpg |
| 2840.jpg | 9181.jpg | 9180.jpg |
| 2870.jpg | 9253.jpg | 9254.jpg |
| 2880.jpg | 9530.jpg | 9520.jpg |
| 7550.jpg | 9584.jpg | 9592.jpg |
| 7620.jpg | 9921.jpg | 9920.jpg |

**Pictures from IAPS selected for the task**
